# Predictors of neonatal mortality: development and validation of prognostic models using prospective data from rural Bangladesh

**DOI:** 10.1136/bmjgh-2019-001983

**Published:** 2020-01-27

**Authors:** Farhad A Khan, Luke C Mullany, Lee F-S Wu, Hasmot Ali, Saijuddin Shaikh, Kelsey Alland, Keith P West Jr, Alain B Labrique

**Affiliations:** Department of International Health, Johns Hopkins Bloomberg School of Public Health, Baltimore, Maryland, USA

**Keywords:** neonatal mortality, newborn mortality, danger sign, symptom, Bangladesh, prognostic model

## Abstract

**Objective:**

To assess the extent to which maternal histories of newborn danger signs independently or combined with birth weight and/or gestational age (GA) can capture and/or predict postsecond day (age>48 hours) neonatal death.

**Methods:**

Data from a cluster-randomised trial conducted in rural Bangladesh were split into development and validation sets. The prompted recall of danger signs and birth weight measurements were collected within 48 hours postchildbirth. Maternally recalled danger signs included cyanosis (any part of the infant’s body was blue at birth), non-cephalic presentation (part other than head came out first at birth), lethargy (weak or no arm/leg movement and/or cry at birth), trouble suckling (infant unable to suckle/feed normally in the 2 days after birth or before death, collected 1-month postpartum or from verbal autopsy). Last menstrual period was collected at maternal enrolment early in pregnancy. Singleton newborns surviving 2 days past childbirth were eligible for analysis. Prognostic multivariable models were developed and internally validated.

**Results:**

Recalling ≥1 sign of lethargy, cyanosis, non-cephalic presentation or trouble suckling identified postsecond day neonatal death with 65.3% sensitivity, 60.8% specificity, 2.1% positive predictive value (PPV) and 99.3% negative predictive value (NPV) in the development set. Requiring either lethargy or weight <2.5 kg identified 89.1% of deaths (at 39.7% specificity, 1.9% PPV and 99.6% NPV) while lethargy or preterm birth (<37 weeks) captured 81.0% of deaths (at 53.6% specificity, 2.3% PPV and 99.5% NPV). A simplified model (birth weight, GA, lethargy, cyanosis, non-cephalic presentation and trouble suckling) predicted death with good discrimination (validation area under the receiver-operator characteristic curve (AUC) 0.80, 95% CI 0.73 to 0.87). A further simplified model (GA, non-cephalic presentation, lethargy, trouble suckling) predicted death with moderate discrimination (validation AUC 0.74, 95% CI 0.66 to 0.81).

**Conclusion:**

Maternally recalled danger signs, coupled to either birth weight or GA, can predict and capture postsecond day neonatal death with high discrimination and sensitivity.

**Key questions**

**What is already known?**

- Neonatal mortality declines in low-income countries have slowed significantly.
- Current approaches to identifying illness in communities heavily rely on infrequent visits by overburdened community health workers; first contact with a trained provider may be long after the period of greatest danger has passed.
- Reaching and assessing newborns in the first week of life can be challenging, but is also likely to have the greatest survival benefit.

**What are the new findings?**

- Neonatal danger signs ascertained through prompted maternal recall shortly after childbirth, if coupled with either birth weight or gestational age (GA), can predict and capture neonatal death with good discrimination and high sensitivity, respectively.

**What do the new findings imply?**

- Given the severe resource limitations experienced in many communities, neonatal illness screening and referral may potentially be improved on the basis of a limited set of predictors which include maternal recall of the newborn’s condition at birth, birth weight and GA.
- Moreover, these findings provide support for systematically capturing GA and birth weight in low-income and middle-income countries.

## Introduction

Despite dramatic reductions in under-five mortality in recent years, progress has largely benefitted infants surviving past the neonatal period, with a marked plateau in neonatal mortality declines.^1^ According to 2015 estimates, 45% of deaths among children under-five occurred in the neonatal period.^2^ As the greatest number of deaths happen during the first hours of life,^1^ there is an urgent need to identify sick newborns and deliver efficacious interventions in a timely manner. However, identifying and treating neonatal illnesses in this window is challenging because neonatal deaths are exclusively concentrated in low-income and middle-income countries with half of these deaths stemming from childbirths occurring in the home,^3^ usually far from skilled obstetric care and where resources to intervene in the event of an emergency are limited.^4 5^

Increasing rates of facility-based births have not resulted in marked improvements in neonatal survival outcomes likely because poor quality of care persists and the onset of complications begins after discharge from the facility.^6^ To ensure high-quality care, processes including systematic assessment and correct diagnosis need to be improved.^7^ Risk assessment tools that evaluate the condition of newborns can aid facility health workers in determining whether intervention is necessary.^8^ Many of these tools are quite sophisticated, requiring skilled clinical personnel or are targeted towards neonatal intensive care units;^9 10^ their need for complex, resource-intensive inputs limits their applicability in low-income settings. Even when births occur in rural facilities, these facilities are often under-resourced and may be unable to conduct routine assessments that require temperature or even weight.^11^ Although a prediction model for neonatal mortality in low-income and middle-income countries was developed recently using surveillance data from sites in India, Nepal and Bangladesh,^12^ alternative models and risk assessment tools may be useful where severe data gaps exist.

At the community level, home-based neonatal care programmes have been used to identify and treat neonatal illnesses.^13 14^ However, attending births and completing postnatal visits in the hours following birth can be challenging for community health workers (CHW),^15^ who are often in short supply and overtaxed with many health provision responsibilities. Although prompt warning sign recognition may potentially translate into timely care seeking and intervention, it is generally difficult for mothers to spontaneously recognise danger signs.^16–19^ In circumstances where danger signs are recognised early, misperceptions regarding severity may inhibit caretakers from seeking timely care.^20 21^ We therefore propose that there is programmatic value in identifying a minimum set of readily observable predictors that can be used to aid neonatal illness screening in the first few highest-risk days of life. However, such a set of predictors would need to capture all infants at risk of dying at high sensitivity, be readily observable or prompted with minimal or no training and could be collected within hours of childbirth.

We sought to assess, through a secondary analysis of a large, prospective dataset from a cluster-randomised trial conducted in Gaibandha and Rangpur, northern Bangladesh, whether the prompted maternal recall of neonatal danger signs (lethargy, cyanosis, non-cephalic presentation and trouble suckling) defined in local terms, could be predictive of postsecond day neonatal death and whether predictive capacity was improved by combining these risk factors with birth weight and gestational age (GA). For this purpose, the aims of this study were to characterise the association between these danger signs and postsecond day neonatal mortality; to describe the sensitivity, specificity, positive predictive value (PPV) and negative predictive value (NPV) at which combinations of these danger signs with GA and birth weight captured mortality and to develop and internally validate prognostic multivariable models using birth weight, GA and maternally reported danger signs as predictors.

## Methods

### Data sources, participants and outcome

**Figure 1:**
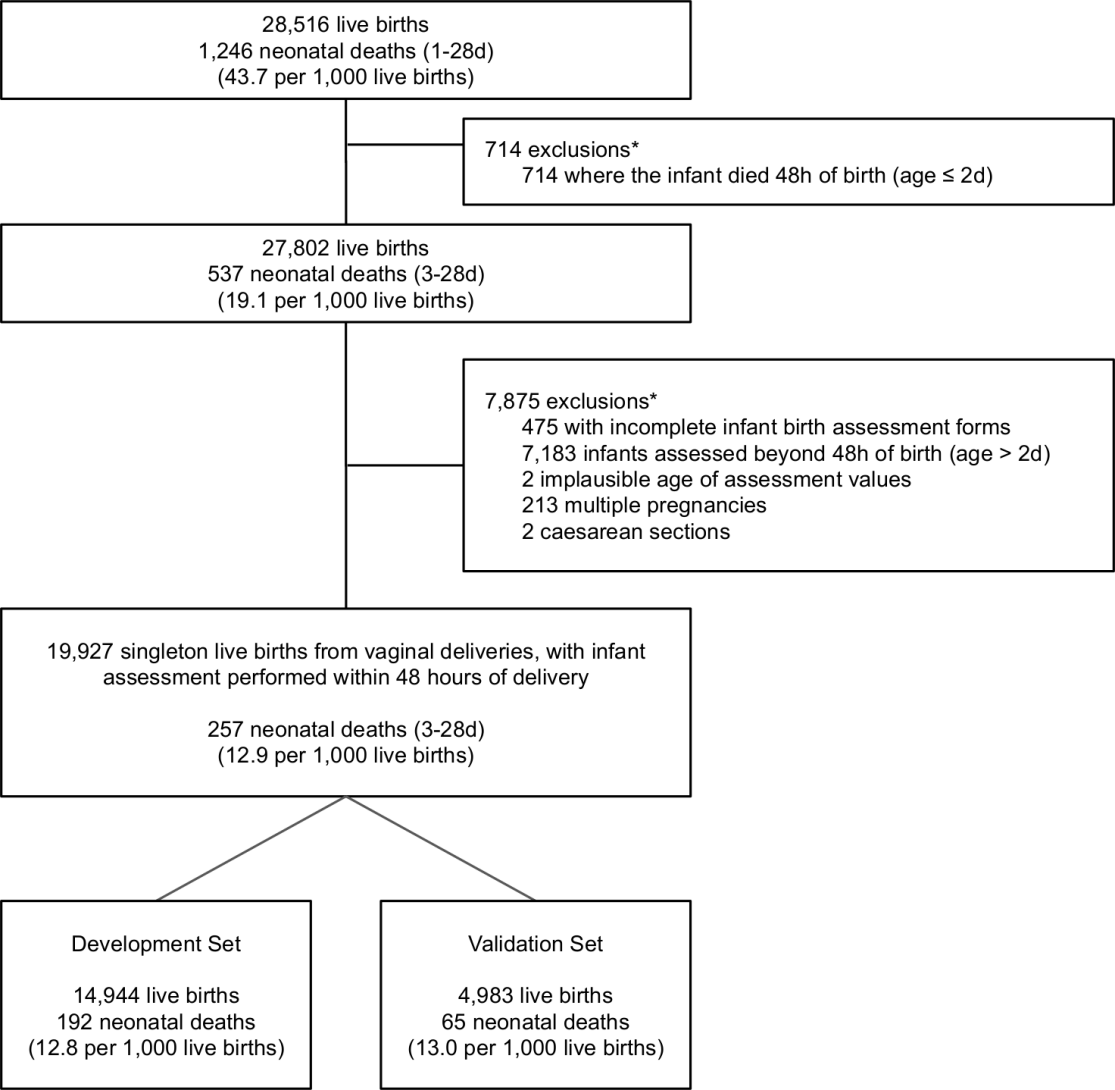
Analytic flow diagram. *Infants may fulfil multiple exclusion criteria.

This is a secondary analysis of the JiVitA-3 cluster-randomised trial dataset, of which the protocol and methods are published elsewhere.^22^ The parent study, which evaluated the efficacy of antenatal micronutrient supplementation versus iron-folate supplementation on all-cause 6-month infant mortality in a community-based setting, was conducted in 596 sectors across 19 unions of Gaibandha and Rangpur districts of northwestern Bangladesh between January 2008 (start of pregnancy surveillance) and August 2012 (end of follow-up). Enrolment of mothers took place early in pregnancy. The population-based cohort enrolled 44 567 pregnant women, with 28 516 live-born infants. Infants were excluded from this analysis if they died within 2 days postbirth (age of death ≤48 hours), if an assessment following birth was absent/incomplete or performed beyond 2 days past birth (age >48 hours), if the age of assessment was implausible (ie, negative), if they were born from a multiple pregnancy, if they were delivered through caesarean section (figure 1). The analytic cohort was then randomly split into a model development (75%) and model validation set (25%), stratified by neonatal deaths, once the final set of eligible records was reached (figure 1).

The outcome of interest, postsecond day neonatal death (age>48 hours), was ascertained for all infants through a 1-month vital status visit, with details captured in an extensive verbal autopsy performed within 30–45 days after an infant death was reported. Few (n=5) infants were lost to follow-up prior to the final 1-month visit, and for this analysis were presumed to have survived. No actions were performed to blind the assessment of the outcome.

### Predictors

An assessment of the infant was performed at home as soon as possible after birth where measurements (birth weight, length, mid-upper arm circumference) were collected by a trained anthropometrist and a simple interview regarding the condition of the infant at birth and newborn care practices was performed.^22^ The mother was asked which part of the baby came out first (head, cord, arm/leg, buttocks, c-section, other, don’t know). Non-cephalic presentation was defined as any part that was not the head coming out first. Cyanosis was defined as any part of the infants body, as reported by the mother, was blue at birth. The strength with which the baby cried and/or moved at birth (either: strong, weak, none) was ascertained. Lethargy was defined in this analysis as the infant either not moving, moving weakly, not crying or crying weakly. Suckling vigour was either captured during a 1-month postpartum assessment or during a verbal autopsy if death occurred first; poor suckling was first defined by the infant being unable to suckle or feed normally in the 2 days after birth during the 1-month postpartum assessment. For infants with missing data from the 1-month assessment, poor suckling was then ascertained from a verbal autopsy in which the mother was asked if the infant was able to suckle or breastfeed normally before death. Home births were defined as births occurring in the home of the mother’s husband, mother/father, neighbour/friend/relative or nurse/family welfare visitor.

Baseline maternal characteristics were collected at the time pregnant women were enrolled into the parent trial. Information included age at pregnancy identification (ie, positive urine test), gravidity, pregnancy history (prior stillbirth, prior abortion, prior miscarriage, prior infant death), anthropometry (height, weight), tobacco exposure (chewed betel nut, chewed tobacco, husband smoking) and education. A living standards index capturing socioeconomic status was calculated from reported household assets and household construction materials by principal components analysis.^23^ Women were categorised as chewing betel nut or tobacco if they reported chewing at least once a week. Husbands were categorised as smokers if pregnant women reported them smoking every day or nearly every day. A woman was considered to have a prior infant death, stillbirth, miscarriage or abortion/menstrual regulation if they reported so as an outcome for any prior pregnancy. A self-reported date of last menstrual period (LMP) was recorded at enrolment and used to estimate GA at delivery. In this site, LMP dates are more precise than usually expected as an ongoing prospective pregnancy surveillance system visits eligible women on a monthly basis to capture amenorrhea.

No actions were performed to blind the assessment of any predictors of the outcome. The parent trial’s allocation of multiple micronutrient or iron-folic acid supplementation was double-masked. However, the parent trial intervention was not a predictor in this analysis.

### Sample size and missing data

No sample size calculations were performed; all available data from the parent trial dataset were used in this analysis. Analyses were performed and models were developed and validated using participants with data available for all predictors (complete case analysis).

### Statistical analysis methods

Maternal and infant baseline characteristics were compared between the development and validation sets. Groups were compared using *χ* ^2^ tests for categorical variables and t-tests for continuous variables. The variables used to model associations were handled as categorical. Associations between risk factors and outcomes were calculated using the development set only. The sensitivity, specificity, PPV and NPV of individual risk factors and combinations of risk factors to classify/predict neonatal death was calculated for infants in the development and validation sets, separately.

For multivariable prediction models, birth weight was modelled as a continuous variable through a linear spline with knot at 1.5 kg, GA was modelled as a continuous variable and all other predictors were modelled as categorical. Birth weight was analysed in kilograms as opposed to grams in order to improve the interpretability of model parameters. Three multivariable models were fitted on the development set. Model coefficients (β) and odds ratio (OR) estimates were calculated using multivariable logistic regression models.

The rationale for developing prediction models for this study was to assess the predictive performance of maternally reported danger signs in relation to birth weight and/or GA. Therefore, models whose variables were selected before modelling were based on demographic factors, common risk factors for adverse pregnancy outcomes observed in the literature (including birth weight and GA) and purposively, the maternally reported danger signs of interest. The first model (Model 1) was based on an expanded set of risk factors for neonatal death: birth weight, GA, lethargy, cyanosis, non-cephalic presentation, trouble suckling, infant sex, maternal age at positive urine test, primigravidae, maternal body mass index (BMI), maternal betel nut chewing, maternal tobacco chewing, husband smoking, maternal education and living standards index (above median). The second model (Model 2) focused on a limited set of risk factors for neonatal distress and death: lethargy, cyanosis, non-cephalic presentation, suckling, GA and birth weight.

The variables for the third model (Model 3) were selected during modelling. This further simplified model (Model 3) is based on a limited set of risk factors that may be approximated when birth weight cannot be measured: GA, lethargy, non-cephalic presentation and suckling. Cyanosis was excluded from Model 3 because its parameter estimate was not statistically significant in multivariable analyses (ie, Model 2). A subgroup analysis was also performed to assess if prior infant loss, prior stillbirth, prior miscarriage or prior abortion/menstrual regulation improved model discrimination among women reporting a previous pregnancy. The predictors in these models are specified in the online supplementary data.

10.1136/bmjgh-2019-001983.supp1 Supplementary data

Model fit (calibration) was assessed with a Hosmer-Lemeshow goodness of fit test, where observations were divided by predicted probability into ten quantiles unless otherwise specified. The degrees of freedom for the Hosmer-Lemeshow test were adjusted for tests on the validation sample. Discrimination was assessed through the area under the receiver-operator characteristic curve (AUC) with asymptotic normal CIs calculated using the DeLong method for estimating standard errors (SEs).^24^ The validation set (25% of the analytical cohort) was used to assess the internal validity of the three models. We largely chose this split ratio to be aligned with common practice. Predicted probabilities were calculated for each observation, using all three models, for observations the validation set. These predictions were then used to calculate discrimination using AUC. Discrimination was compared across all three models.

All statistical tests were two-sided. All analyses were performed using Stata 15 (Stata, College Station, Texas, USA).

### Informed consent, ethical approval and trial registration

Once identified as pregnant from a population-based surveillance, oral consent to participate in the parent trial was obtained from pregnant women before a witness. The parent trial was approved by the Institutional Review Board at the Johns Hopkins Bloomberg School of Public Health (Baltimore, Maryland, USA) under registration number IRB00000570 and the Bangladesh Medical Research Council (Dhaka, Bangladesh). The parent trial is registered at clinicaltrials.gov (Identifier: NCT00860470). This reporting of this analysis followed the transparent reporting of a multivariable prediction model for individual prognosis or diagnosis (TRIPOD) guidelines (online supplementary data, table S1).^25 26^

### Patient and public involvement

Patients and/or the general public were not involved in the design, execution or drafting of this secondary analysis.

## Results

### Study population

The cohort of infants in the parent trial consisted of 28 516 live births including 1246 neonatal deaths (figure 1). Excluding 714 infants that did not survive 2 days past childbirth resulted in an analytic cohort with a postsecond day neonatal mortality rate of 19.1 deaths per 1000 live births. After all exclusion criteria were applied, the cohort was randomly split into development and validation sets. The development set included 192 neonatal deaths among 14 944 live births (12.8 postsecond day neonatal deaths per 1000 live births) and the validation set included 65 neonatal deaths among 4983 live births (13.0 postsecond day neonatal deaths per 1000 live births) (figure 1). In the development set, infants that died during the postsecond day neonatal period differed from those that survived with respect to the following characteristics: gravidity, maternal BMI<18.5 kg/m^2^, living standards index, experience of an prior infant death (among multigravidae), location of delivery, GA at birth, birth weight, cyanosis, non-cephalic presentation, lethargy and trouble suckling (table 1). A similar comparison was made for the validation set, in which cyanosis and lethargy did not differ among infants that died versus infants that survived (online supplementary data, table S2). The development and validation sets appeared to be balanced with respect to baseline maternal and infant demographic characteristics (online supplementary data, table S3). There was a slight imbalance in infant sex in the validation set, where 52.3% of infants were male and 47.7% of infants were female. The median age of death was 5 days (IQR: 3–12) in the development set and 6 days in the validation set (IQR: 3–11) where age at birth was defined as 1 day. The median time of assessment was 11 hours after birth (IQR 6–18) in both the development and the validation sets.

**Table 1:** Maternal and newborn infant characteristics stratified by postsecond day neonatal survival in the development set*

	Development set (n=14 944)				
	Alive (n=14 752)		Dead (n=192)		P value
	No.	%	No.	%	
Age at positive urine test, years					
<20	4521	30.6	71	37.0	0.154
20–29	8197	55.6	95	49.5	
>29	2034	13.8	26	13.5	
Missing	0	0.0	0	0.0	
Gravidity					
Multigravidae	10 438	70.8	112	58.3	<0.001
Primigravidae	4314	29.2	80	41.7	
Missing	0	0.0	0	0.0	
Maternal BMI, kg/m^2^					
BMI≥18.5	8755	59.3	100	52.1	0.042
BMI<18.5	5997	40.7	92	47.9	
Missing	0	0.0	0	0.0	
Living standards index†					
At/below median	7336	49.7	123	64.1	<0.001
Above median	7394	50.1	69	35.9	
Missing	22	0.1	0	0.0	
Education level					
None	4218	28.6	69	35.9	0.103
Class 1–4	2215	15	33	17.2	
Class 5–9	7496	50.8	83	43.2	
Class ≥10	803	5.4	7	3.6	
Missing	20	0.1	0	0.0	
Betel nut chewing‡					
Did not chew	4524	30.7	60	31.2	0.762
Chewed betel nut	10 188	69.1	132	68.8	
Missing	40	0.3	0	0.0	
Tobacco chewing‡					
Did not chew	12 998	88.1	173	90.1	0.521
Tobacco chewing	1713	11.6	18	9.4	
Missing	41	0.3	1	0.5	
Husband smoking‡					
Did not smoke	5109	34.6	71	37.0	0.582
Husband smoking	9592	65	121	63.0	
Missing	51	0.3	0	0.0	
Experienced prior stillbirth§					
No prior stillbirth	9541	91.6	97	86.6	0.172
≥1 previous stillbirth	879	8.4	15	13.4	
Missing	1	0.0	0	0.0	
Experienced prior abortion§					
No prior abortion	9232	88.6	99	88.4	0.992
≥1 prior abortion	1188	11.4	13	11.6	
Missing	1	0.0	0	0.0	
Experienced prior miscarriage§					
No prior miscarriage	9275	89	98	87.5	0.874
≥1 prior miscarriage	1145	11	14	12.5	
Missing	1	0.0	0	0.0	
Experienced prior infant death§					
No prior infant death	7722	74.1	65	58.0	0.001
≥1 prior infant death	2222	21.3	38	33.9	
Missing	477	4.6	9	8.0	
Location of delivery¶					
Facility	982	6.7	7	3.6	0.007
Home	13 743	93.2	183	95.3	
Missing	27	0.2	2	1.0	
Infant sex¶					
Male	7481	50.7	98	51.0	0.928
Female	7271	49.3	94	49.0	
Missing	0	0.0	0	0.0	
Gestational age at birth, weeks					
Mean (SD)	38.8 (2.86)		36.2 (4.19)		<0.001
≥37	11 424	77.4	85	44.3	<0.001
<37	2690	18.2	100	52.1	
Missing	638	4.3	7	3.6	
Birth weight, kg¶					
Mean (SD)	2.57 (0.40)		2.04 (0.58)		<0.001
≥2.5	8542	57.9	49	25.5	<0.001
<2.5	6177	41.9	140	72.9	
Missing	33	0.2	3	1.6	
Cyanosis¶					
Absent	14 396	97.6	183	95.3	0.034
Present	308	2.1	9	4.7	
Missing	48	0.3	0	0.0	
Non-cephalic presentation¶					
Absent	14 439	97.9	180	93.8	<0.001
Present	297	2.0	12	6.2	
Missing	16	0.1	0	0.0	
Lethargy¶					
Absent	9805	66.5	88	45.8	<0.001
Present	4854	32.9	104	54.2	
Missing	93	0.6	0	0.0	
Trouble suckling					
Absent	13 438	91.1	128	66.7	<0.001
Present	1171	7.9	54	28.1	
Missing	143	1.0	10	5.2	

*Data are n, % unless otherwise specified.

†Median living standards index for combined development and validation sets: –0.2596556.

‡Tobacco exposures measured with respect to the week preceding interview at enrolment.

§Data ascertained from pregnancy enrolment among women reporting a previous pregnancy. In the development set, 10 533 infants were born to mothers in multigravidae, 10 421 remained alive, 112 died.

¶Data ascertained from infant birth assessment or maternal birth assessment.

BMI, body mass index.

### Association between maternally reported danger signs and neonatal mortality

The association between maternally reported danger signs (lethargy, cyanosis, non-cephalic presentation, trouble suckling), measured predictors (birth weight, GA) and neonatal death was assessed for the development set. Each of the maternally reported danger signs was associated with mortality in bivariate analyses (table 2). However, in a multivariable model, the association between cyanosis and neonatal mortality was imprecise (OR 1.10; 95% CI 0.54 to 2.24). The associations between neonatal death and lethargy (OR 1.51, 95% CI 1.15 to 1.98), non-cephalic presentation (OR 2.59, 95% CI 1.49 to 4.48) and trouble suckling (OR 3.47, 95% CI 2.57 to 4.70) remained strong in multivariable analyses (table 2). Birth weight (<2500 g, <2000 g, <1500 g) and GA<37 weeks were associated with neonatal mortality in both bivariate and multivariable analyses (table 2). An increase in the magnitude of association was also observed as the number of danger signs reported for a given infant increased. In a multivariable model, the OR (95% CI) of neonatal death for infants presenting one, two and three danger signs was 1.64 (1.21 to 2.21), 4.33 (2.97 to 6.32) and 8.86 (4.35 to 18.1) compared with infants presenting no danger signs, respectively (table 2). Few deaths were observed for cyanotic newborns (9 out of 317) and for newborns presenting non-cephalically (12 out of 309) in the development set (table 2).

**Table 2:** Associations between maternally-reported danger signs, birth weight, gestational age and neonatal mortality

	Infants (n=14 944)	Deaths (n=192)	Mortality rate*	Univariate OR (95% CI)	Multivariable† OR (95% CI)
Individual predictors					
Lethargy‡	4958	104	20.98	2.16 (1.69 to 2.76)	1.51 (1.15 to 1.98)
Cyanosis‡	317	9	28.39	2.40 (1.36 to 4.23)	1.10 (0.54 to 2.24)
Non-cephalic presentation‡	309	12	38.83	3.47 (2.10 to 5.73)	2.59 (1.49 to 4.48)
Trouble suckling‡	1225	54	44.08	5.07 (3.84 to 6.69)	3.47 (2.57 to 4.70)
Birth weight‡					
<2.5 kg	6317	140	22.16	4.05 (3.05 to 5.38)	2.67 (1.95 to 3.64)
<2.0 kg§	1172	91	77.65	12.1 (9.4 to 15.5)	6.46 (4.79 to 8.73)
<1.5 kg§	110	36	327.27	50.8 (35.3 to 73.0)	16.7 (10.8 to 25.7)
Gestational age‡					
<37 weeks	2790	100	35.84	4.87 (3.78 to 6.28)	3.58 (2.72 to 4.71)
Number of reported danger signs					
None	8908	66	7.41	REF	REF
One	4839	70	14.47	1.85 (1.38 to 2.47)	1.64 (1.21 to 2.21)
Two	820	37	45.12	6.23 (4.39 to 8.85)	4.33 (2.97 to 6.32)
Three	75	9	120.0	16.2 (8.36 to 31.3)	8.86 (4.35 to 18.1)
Four	3	0	NA	NA	NA

*Mortality rate expressed in deaths per 1000 live births.

†Multivariable models for individual predictors include lethargy, cyanosis, malpresentation, poor suckling, birth weight < 2.5 g and gestational age <37 weeks.

‡Missing data on lethargy for 93 infants (0 of which died), cyanosis for 48 infants (of which 0 died), non-cephalic presentation for 16 infants (in which 0 died), trouble suckling for 153 infants (of which 10 died), birth weight for 36 infants (3 of which died) and gestational age for 638 infants (7 of whom died).

§Multivariable model includes birth weight <1.5 kg or <2.0 kg in lieu of birth weight <2.5 kg.

### Classification and multivariable prediction models

The sensitivity, specificity, PPV and NPV of neonatal death were calculated for each risk factor and combinations of risk factors (table 3). The PPV for lethargy, cyanosis, non-cephalic presentation and trouble suckling did not exceed 6% in either the development or validation sets. However, lethargy alone classified neonatal deaths at 54.2% sensitivity in the development set and adding at least one recalled sign of cyanosis, non-cephalic presentation or trouble suckling to lethargy increased the sensitivity to 65.3% in the development set (table 3). A notable increase in the sensitivity of predictor combinations was observed when low birth weight (<2.5 kg) was added to combinations of maternally recalled danger signs. A combination of either lethargy or birth weight <2.5 kg classified neonatal deaths with 89.1% sensitivity (39.7% specificity, 1.9% PPV and 99.6% NPV) in the development set and 84.6% sensitivity in the validation set (table 3). Low birth weight (birth weight <2.5 kg) alone captured deaths at 74.1% sensitivity, 58.0% specificity, 2.2% PPV and 99.4% NPV (table 3). A combination of either lethargy or GA<37 weeks classified neonatal deaths with 81.0% sensitivity (53.6% specificity, 2.3% PPV and 99.5% NPV) in the development set and 71.4% sensitivity in the validation set (table 3). Preterm birth alone (GA<37 weeks) captured deaths at 54.1% sensitivity, 80.9% specificity, 3.6% PPV and 99.3% NPV (table 3). Because the PPV observed among the more sensitive predictor combinations did not generally exceed 5%, we examined whether more specific or restrictive combinations of predictors could result in increases in PPV. Reporting both lethargy and birth weight <2.0 kg captured infants at 25.9% sensitivity, 96.9% specificity and predicted deaths at 9.6% PPV (table 3).

**Table 3:** Sensitivity, specificity, PPV and NPV for individual and combined predictors of neonatal death

	Development set (n=14 944)*				Validation set (n=4983)*			
	Sensitivity (%)	Specificity (%)	PPV (%)	NPV (%)	Sensitivity (%)	Specificity (%)	PPV (%)	NPV (%)
Individual predictors								
Lethargy	54.2	66.9	2.1	99.1	44.6	66.5	1.7	98.9
Cyanosis	4.7	97.9	2.8	98.7	6.2	97.6	3.3	98.7
Non-cephalic presentation	6.3	98.0	3.9	98.8	7.7	98.0	5.0	98.8
Poor suckling	29.7	92.0	4.4	99.1	32.8	92.2	5.0	99.1
Birth weight								
<2.5 kg	74.1	58.0	2.2	99.4	75.4	58.7	2.4	99.4
<2.0 kg	48.1	92.7	7.8	99.3	49.2	93.2	8.7	99.3
<1.5 kg	19.0	99.5	32.7	99.0	24.6	99.5	39.0	99.0
Gestational age								
<37 weeks	54.1	80.9	3.6	99.3	51.7	80.8	3.2	99.3
Predictor combinations								
≥1 of lethargy, cyanosis or non-cephalic presentation or trouble suckling	65.3	60.8	2.1	99.3	61.5	60.4	2.0	99.2
≥1 of lethargy, cyanosis or non-cephalic presentation	56.8	65.8	2.1	99.1	50.8	64.9	1.9	99.0
≥1 of lethargy, trouble suckling or birth weight <2.5 kg	90.1	37.1	1.8	99.7	86.2	37.5	1.8	99.5
≥1 of lethargy or birth weight <2.5 kg	89.1	39.7	1.9	99.6	84.6	40.1	1.8	99.5
Lethargy and birth weight <2.5 kg	38.6	85.2	3.3	99.1	35.4	85.0	3.0	99.0
≥1 of lethargy or birth weight <2.0 kg	76.0	62.6	2.6	99.5	63.1	62.6	2.2	99.2
Lethargy and birth weight <2.0 kg	25.9	96.9	9.6	99.0	30.8	97.0	12.1	99.1
≥1 of lethargy or gestational age <37 weeks	81.0	53.6	2.3	99.5	71.4	53.3	2.0	99.3
≥1 of birth weight <2.5 kg or gestational age <37 weeks	81.4	49.4	2.1	99.5	82.5	49.4	2.1	99.5

*Missing values resulting in development and validation sets, respectively, of sizes: 14 851 and 4959 for lethargy; 14 896 and 4959 for cyanosis; 14 928 and 4981 for non-cephalic presentation; 14 791 and 4925 for trouble suckling; 14 908 and 4967 for birth weight (<2.5 kg, <2.0 kg, <1.5 kg), 14 299 and 4718 for gestational age <37 weeks; 14 729 and 4917 for ≥1 of lethargy, cyanosis, non-cephalic presentation or trouble suckling; 14 826 and 4952 for ≥1 of lethargy, cyanosis non-cephalic presentation, 14 832 and 4934 for ≥1 of lethargy, trouble suckling or birth weight <2.5 kg; 14 882 and 4956 for ≥1 of lethargy or birth weight <2.5 kg; 14 877 and 4970 for lethargy and birth weight <2.5 kg; 14 845 and 4952 for ≥1 of lethargy or birth weight <2.0 kg; 14 914 and 4974 for lethargy and birth weight <2.0 kg; 14 439 and 4831 for ≥1 of lethargy or gestational age. <37 weeks and 14 493 and 4828 for ≥1 of birth weight <2.5 kg or gestational age <37 weeks.

NPV, negative predictive value; PPV, positive predictive value.

Three multivariable prediction models were developed; model fit and discrimination were compared across development and validation sets. All models fit the data across development and validation sets (Hosmer-Lemeshow p>0.05). Model 1 (expanded set of risk factors) predicted neonatal death with good discrimination in the development set (AUC 0.81, 95% CI 0.77 to 0.84) and the validation set (AUC 0.80, 95% CI 0.73 to 0.88) (table 4). Model 2 (limited set of risk factors for neonatal distress and death) predicting neonatal death with good discrimination in the development set (AUC 0.80, 95% CI 0.76 to 0.84) and in the validation set (AUC 0.80, 95% CI 0.73 to 0.87), performed as well as Model 1 (table 4). Model 3 (GA, lethargy, trouble suckling and non-cephalic presentation) retained moderate discrimination in both the development set (AUC 0.75, 95% CI 0.71 to 0.80) and the validation set (AUC 0.74, 95% CI 0.66 to 0.81) given the exclusion of birth weight (table 4). Receiver-operator characteristic curves and AUC were produced for the three models (online supplementary data, figure S1). A subgroup analysis among multigravidae revealed that prior infant loss, previous abortion, previous miscarriage and previous stillbirth did not increase discrimination beyond birth weight, GA, non-cephalic presentation, lethargy, cyanosis and trouble suckling (online supplementary data, table S4). A individual newborn’s predicted probability of death in the neonatal period can be calculated using the provided risk equations for Models 1 and 2 (online supplementary data, table S5).

**Table 4:** Prediction models for postsecond day neonatal mortality, model discrimination and calibration

	Univariate	Model 1		Model 2		Model 3	
Total number of newborns		13 932		13 985		14 056	
Number of newborn deaths		171		172		175	
Predictors	**OR (95% CI**)	**OR (95% CI**)	**β**	**OR (95% CI**)	**β**	**OR (95% CI**)	**β**
Birth weight, kg*							
≤1.5	0.00 (0.00 to 0.02)	0.01 (0.00 to 0.09)	−4.413	0.01 (0.00 to 0.06)	−4.853		
>1.5	0.08 (0.06 to 0.13)	0.16 (0.10 to 0.25)	−1.834	0.15 (0.10 to 0.23)	−1.904		
Gestational age, weeks	0.81 (0.78 to 0.83)	0.91 (0.86 to 0.95)	−0.100	0.91 (0.87 to 0.95)	−0.097	0.82 (0.79 to 0.85)	−0.203
Lethargy	2.39 (1.79 to 3.18)	1.54 (1.11 to 2.14)	0.431	1.53 (1.11 to 2.12)	0.426	1.95 (1.43 to 2.66)	0.668
Cyanosis	2.30 (1.17 to 4.53)	1.41 (0.62 to 3.21)	0.340	1.39 (0.61 to 3.15)	0.326		
Non-cephalic presentation	3.24 (1.79 to 5.88)	2.16 (1.12 to 4.17)	0.771	2.07 (1.08 to 3.97)	0.726	2.47 (1.32 to 4.61)	0.904
Poor suckling	4.84 (3.50 to 6.69)	2.64 (1.81 to 3.83)	0.969	2.58 (1.78 to 3.74)	0.947	3.98 (2.84 to 5.58)	1.381
Female sex	0.99 (0.74 to 1.31)	0.95 (0.69 to 1.31)	−0.049				
Maternal age at urine test, years							
<18	REF	REF					
20–29	0.74 (0.54 to 1.01)	1.20 (0.76 to 1.88)	0.180				
>29	0.81 (0.52 to 1.28)	1.20 (0.62 to 2.33)	0.186				
Primigravidae	1.73 (1.29 to 2.31)	1.33 (0.85 to 2.10)	0.288				
Maternal BMI <18.5 kg/m^2^	1.34 (1.01 to 1.79)	1.20 (0.87 to 1.66)	0.186				
Betel nut chewing	0.98 (0.72 to 1.33)	1.12 (0.79 to 1.60)	0.116				
Tobacco chewing	0.79 (0.48 to 1.29)	0.71 (0.40 to 1.26)	−0.344				
Husband smoking	0.91 (0.68 to 1.22)	0.83 (0.59 to 1.16)	−0.192				
Education level							
No education	REF	REF					
Class 1–4	0.91 (0.60 to 1.38)	0.91 (0.57 to 1.47)	−0.092				
Class 5–9	0.68 (0.49 to 0.93)	0.79 (0.52 to 1.18)	−0.241				
Class ≥10	0.53 (0.24 to 1.16)	1.13 (0.45 to 2.79)	0.118				
Living standards index, above median	0.56 (0.41 to 0.75)	0.65 (0.45 to 0.93)	−0.432				
Intercept			7.310		7.787		2.707
Discrimination and calibration							
Area under ROC curve (95% CI)							
Derivation		0.81 (0.77 to 0.84)		0.80 (0.76 to 0.84)		0.75 (0.71 to 0.80)	
Validation		0.80 (0.73 to 0.88)		0.80 (0.73 to 0.87)		0.74 (0.66 to 0.81)	
Hosmer Lemeshow χ2(p)							
Derivation		10.61 (0.22)		6.28 (0.62)		10.25 (0.25)	
Validation		11.59 (0.31)		6.56 (0.77)		10.12 (0.43)	

*Birth weight modelled as a continuous linear spline with a knot at 1.5 kg. Both spline terms were included in the univariate analysis.

## Discussion

### Summary of main findings

This analysis provides evidence on the ability of maternally recalled danger signs (lethargy, cyanosis, non-cephalic presentation, trouble suckling), birth weight and GA to predict postsecond day neonatal mortality. It is important to note that for maternally recalled danger signs mothers were neither trained nor standardised in the detection or reporting of these signs, but that these findings are based on over 19 000 such reports captured in a typical rural setting in Bangladesh.^27^ Associations were observed between these danger signs and neonatal death in bivariate analyses. with the exception of cyanosis (maternal recall that any part of the baby’s body was blue at birth), these associations retained their statistical significance in multivariable, adjusted analyses. Moreover, the magnitude of association increased as the number of reported danger signs increased. These maternally recalled signs, however, did not capture subsequent neonatal death with high sensitivity when modelled on their own. Recalling at least lethargy with at least one sign of cyanosis, non-cephalic presentation or trouble suckling in the development cohort classified neonatal death at 65% sensitivity. In contrast, a decision rule that classified neonatal death for infants who were either lethargic or weighed less than 2.5 kg at birth increased sensitivity to 89% in the development cohort. The specificity of this decision rule was 40% (with 60% of all infants surviving the neonatal period being either lethargic or weighing less than 2.5 kg). Multivariable prediction models that were developed using a development set that included danger signs together with birth weight and/or GA predicted neonatal death in the validation set achieved good discrimination. These findings are consistent with the literature indicating that low birth weight and preterm birth are strong risk factors for neonatal death.^1^

### Implications for public health practice

Predictive models of likely adverse pregnancy outcomes or increased propensity for neonatal distress, prior to the onset of clinical symptoms, have been highly sought after for decades. Scores which discriminate healthy neonates from those at higher risk of early newborn illness, susceptibility to infection or failure to thrive have been attempted—often with variable performance, depending on the skill of the observer and the context of use. The Apgar score is among the most-used metrics for newborn assessments, with demonstrated predictive reliability in clinical settings, captured by trained clinical staff at 1 and 5 min postnatally.^28^ However, in low-resource community settings, where neonatal mortality remains high, where improvement in this critical development metric has stagnated and where achieving full coverage of institutional deliveries is a long-term proposition, innovation in triaging limited resources to high-risk neonates is required. In this rural Bangladesh context, where home-based delivery still occurs 69% of the time,^29^ and where even facility-deliveries are discharged shortly after birth, contact with qualified healthcare providers in the highest risk postnatal period is rare. Although home-based neonatal care packages have emerged as a remedy for these coverage gaps, ensuring that all infants receive timely postnatal care visits still remains a challenge under both controlled and programmatic contexts.^15 30^ Despite a growing body of evidence that suggests early CHW screening can significantly improve outcomes—from early initiation of breastfeeding to thermal care and improved care-seeking behaviours,^30 31^ it is clear that the current availability of health workers in low-level facilities and community health workers hinders their ability to recognise the currently prioritised warning signs.

These challenges underscore the importance of seeking a minimum set of maternally observable danger signs which can reliably distinguish neonates at high risk of mortality. While efforts are made to improve the coverage of institutional delivery and postnatal care in the first week of life, these findings suggest that maternally reportable indicators are both assessable and could reduce preventable neonatal mortality in these settings in the interim. Specifically, our findings help to identify a minimum set of key danger signs worthy of triggering referral that can be highlighted in a late-in pregnancy antenatal care visit or that can be used to counsel recently delivered women and/or their family members, given infrequent CHW postnatal visits. For deliveries that occur in the home, caretakers are generally advised to seek immediate, qualified care from a CHW or health centre if they recognise danger signs. However, given the challenges associated with spontaneous warning sign recognition, caretakers recall may need to be prompted. A recent study in this population demonstrated that a mobile health package of short message service and home visits was a cost-effective addition to census enumeration and pregnancy surveillance. Such a package could potentially be leveraged to prompt recall of these danger signs.^32^

Concerns about maternal capacity to recognise a newborn in danger, nonetheless, exist. Maternal recall of lethargy, non-cephalic presentation or robust suckling have been shown in this data to be reliable, despite likely variability in maternal interpretation of their own child’s condition. Coupling these perceived conditions to information about GA or birth weight could provide a powerful risk index to trigger a CHW home visit or immediate care seeking by the family. Although the PPV of individual and combined danger signs (ranging from 1% to 10%) may be clinically relevant given the low absolute risk of mortality in this population, the PPV of these signs may seem too low to capture deaths in the context of a standalone, one-time screening test. However, we do not see this as problematic. A programmatic approach that aims for high sensitivity, ensuring that no babies at risk of dying are missed while helping overburdened health workers target their resources and time, can be acceptable despite the loss of specificity, but only if it is followed by a more specific screening test or examination on referral. Any infants in the community that are falsely classified at risk of death due to both a loss of sensitivity and low absolute risk of mortality could therefore be introduced into the continuum of care; intervened on in ways that may yield benefits beyond immediate survival (ie, vital registration, vaccination scheduling, postnatal counselling). This approach may be acceptable because the NPV exceeds 98%, suggesting that most infants that screen negative will survive the neonatal period.

Maternal histories of danger signs can be used to support a highly sensitive first test in a two-stage sequential screening process if birth weight and/or GA measurements are available. Because countries with the highest burden of birth weight tend to also lack reliable birth weight data,^33^ efforts are needed to systematically measure and record GA in the long-term (as programmes aim for full coverage of facility-based deliveries) as well as the short-term. Capturing birth weight will be increasingly feasible in low-income and middle-income settings as the cost of robust, portable weighing scales drops with time and as investments are committed towards strengthening health information systems through digital tools.^34^ However, for circumstances in which measuring birth weight may be cost prohibitive, our findings suggest that an estimate of GA can serve as a reasonable alternative. The capture of LMP can be used to estimate GA; our group has previously validated this method as a reliable approach to determining preterm status in this community.^35^ The deployment of such a preliminary screener would likely be by phone, dramatically altering the cost-benefit calculus since mobile birth notification with GA capture, followed by a mobile short interview to assess risk, is increasingly feasible across most of rural Bangladesh and South Asia given trends in mobile phone ownership.^36^

For facilities with the resources to ascertain valid birth weight and GA measurements, a model that also includes lethargy, cyanosis, non-cephalic presentation and trouble suckling will be able to discriminate infants as well as a model with other risk factors for neonatal mortality among primigravidae and multigravidae. As the quality of care in lower-level facilities increases with time and infants are triaged on the basis of more sophisticated risk assessment tools, the predictors we present may potentially be used for purposes other than immediate intervention. For example, these predictors may be used to flag infants for additional monitoring before discharge. If discharged, community health workers may be instructed to selectively follow-up with infants born preterm, with low birth weight or presenting as lethargic on delivery.

### Study strengths and limitations

The strengths of this analysis include a large sample size and complete follow-up for infant outcomes in a rigorous research environment, ensuring high-quality data capture. The risk of recall bias and misclassification was reduced because the analytic sample was restricted to infants assessed either on the date of delivery or the day after. Assessments were performed before infant deaths and the predictors selected for analysis were expressly chosen due to their easy observability. There is a risk of recall bias for poor suckling because it was collected either at a 1-month postpartum assessment or during a verbal autopsy. Other predictors for neonatal illness that could be ascertained through maternal report such as temperature, fast breathing and severe chest indrawing,^37^ were not measured during assessment. The maternally reported predictors that were measured were not standardised or externally validated—although we consider this an important source of natural variation expected in a self-reported score; as mentioned earlier, this included variability enhances the generalisability of these findings. Although maternal recall was prompted in these data, other studies report poor caregiver knowledge or spontaneous recall of danger signs for neonatal death.^16 19^

Although the risk of recurrence of adverse birth outcomes has been previously reported for stillbirth,^38^ low birth weight,^39^ small-for-GA,^40^ and perinatal mortality,^41^ these results indicate that experiencing a prior infant death, previous miscarriage, previous abortion or previous stillbirth do not increase predictive discrimination beyond birth weight, GA and maternally recalled risk factors. The inability of these risk factors to discriminate mortality in these data may be driven by their relatively low prevalence in this study population.

A higher proportion of infants that died during the neonatal period were not assessed on the day of their birth or the day after (within age ≤48 hours) compared with those infants that survived the neonatal period. Although we attempted to mitigate this survivorship bias by restricting any inferences drawn to those infants that survived past 48 hours following childbirth, the postsecond day mortality rate in the development set was 19 deaths per 1000 live births before exclusions and 13 deaths per 1000 live births after exclusions. We speculate that this bias attenuated model coefficient estimates because neonatal deaths were differentially depleted from the study population.

Nevertheless, the most important limitation of this analysis is that it attempts to use predictors that are probably most useful at the point of delivery (non-cephalic presentation, cyanosis) to predict mortality during the period of time at which they may be less relevant, past 48 hours postbirth. Non-cephalic presentation, for example, is strongly associated with birth asphyxia and stillbirth.^42 43^ Few deaths were observed beyond the intrapartum period among infants who had presented non-cephalically or among infants cyanotic at birth. Late neonatal deaths, however, are predominantly caused by preterm birth (21%) or infection (48%) where infection is further divided into sepsis (37%), pneumonia (5%) and diarrhoea (2%).^1^ The general symptoms for neonatal sepsis include fever, temperature instability, ‘not doing well’.^44^

Furthermore, the use of split sample validation as opposed to cross-validation or temporal validation as the method for internal validation, Hosmer-Lemeshow tests for the calibration of a model with a binary outcome, the relatively few events per predictor, and the absence of imputation methods for predictors are major weaknesses for the model’s internal validity. However, the purpose of developing prediction models was to assess how well maternally observed danger signs, in addition to birth weight and/or GA, discriminates infants. Given these results, we would recommend that these data be used to inform the future development of prediction models that prospectively capture and assess how maternally reported danger signs may improve discrimination among all neonates (including outcomes occurring within 48 hours of childbirth). Moreover, the generalisability and programmatic applications of these predictors and models will ultimately depend on their validation in other populations.

## Conclusion

Maternal histories of lethargy, non-cephalic presentation and trouble suckling in their newborn shortly after birth were, in this rural Bangladeshi setting, reasonably predictive of postsecond day neonatal death, but only if coupled to a measurement of birth weight and GA. These findings may be used to support efforts to triage and focus care to high-risk newborns, to advocate for the systematic capture of birth weight and GA at birth to improve prediction and ultimately, to reduce preventable neonatal mortality in resource-constrained settings. As Bangladesh and neighbouring countries struggle to cover existing workforce gaps, strategies which enable resource prioritisation and target care to those at greatest risk may be useful in accelerating reductions in neonatal mortality.
