# Supplementary material for: Predictors of neonatal mortality: development and validation of prognostic models using prospective data from rural Bangladesh
Source: BMJ Glob Health. 2020 Jan 27;5(1):e001983. doi: 10.1136/bmjgh-2019-001983 (PMC7042570; doi:10.1136/bmjgh-2019-001983)
Supplement: Supplementary data [file bmjgh-2019-001983supp001.pdf]

SUPPLEMENTARY DATA

Methods

The effect of multigravid risk factors was explored through a series of models. Model A is based on the following risk factors: birthweight, gestational age, lethargy, cyanosis, non-cephalic presentation, poor suckling, ≥1 prior abortion, ≥1 prior miscarriage, ≥1 prior stillbirth, and ≥1 prior infant death. Model B is based on the following risk factors: birthweight, gestational age, lethargy, cyanosis, non-cephalic presentation, poor suckling. Model C is based on the following risk factors: gestational age, lethargy, non-cephalic presentation, and poor suckling. Model D is based on the following risk factors: ≥1 prior abortion, ≥1 prior miscarriage, ≥1 prior stillbirth, and ≥1 prior infant death.

Table S1: Transparent reporting of a multivariable prediction model for individual prognosis or diagnosis (TRIPOD) checklist[1,2]

Section/Topic	Item		Checklist Item	Manuscript Section
Title and abstract				
Title	1	D;V	Identify the study as developing and/or validating a multivariable prediction model, the target population, and the outcome to be predicted.	Title
Abstract	2	D;V	Provide a summary of objectives, study design, setting, participants, sample size, predictors, outcome, statistical analysis, results, and conclusions.	Abstract
**Introduction**				
Background and objectives	3a	D;V	Explain the medical context (including whether diagnostic or prognostic) and rationale for developing or validating the multivariable prediction model, including references to existing models.	Intro; para 1-3
	3b	D;V	Specify the objectives, including whether the study describes the development or validation of the model or both.	Intro; para 4
**Methods**				
Source of data	4a	D;V	Describe the study design or source of data (e.g., randomized trial, cohort, or registry data), separately for the development and validation data sets, if applicable.	Methods; data sources, participants, and outcome, para 1
	4b	D;V	Specify the key study dates, including start of accrual; end of accrual; and, if applicable, end of follow-up.	
Participants	5a	D;V	Specify key elements of the study setting (e.g., primary care, secondary care, general population) including number and location of centres.	
	5b	D;V	Describe eligibility criteria for participants.	
	5c	D;V	Give details of treatments received, if relevant.	
Outcome	6a	D;V	Clearly define the outcome that is predicted by the prediction model, including how and when assessed.	Methods; data sources, participants, and outcome, para 2
	6b	D;V	Report any actions to blind assessment of the outcome to be predicted.	
Predictors	7a	D;V	Clearly define all predictors used in developing or validating the multivariable prediction model, including how and when they were measured.	Methods; predictors, para 1-2
	7b	D;V	Report any actions to blind assessment of predictors for the outcome and other predictors.	Methods; predictors, para 3
Sample size	8	D;V	Explain how the study size was arrived at.	Methods; sample size and missing data, para 1
Missing data	9	D;V	Describe how missing data were handled (e.g., complete-case analysis, single imputation, multiple imputation) with details of any imputation method.	
Statistical analysis methods	10a	D	Describe how predictors were handled in the analyses.	Methods; statistical analysis methods, para 1-2
	10b	D	Specify type of model, all model-building procedures (including any predictor selection), and method for internal validation.	Methods; statistical analysis methods, para 3-5
	10c	V	For validation, describe how the predictions were calculated.	Methods; statistical analysis methods, para 5
	10d	D;V	Specify all measures used to assess model performance and, if relevant, to compare multiple models.	
	10e	V	Describe any model updating (e.g., recalibration) arising from the validation, if done.	NA
Risk groups	11	D;V	Provide details on how risk groups were created, if done.	NA
Development vs. validation	12	V	For validation, identify any differences from the development data in setting, eligibility criteria, outcome, and predictors.	NA
**Results**				
Participants	13a	D;V	Describe the flow of participants through the study, including the number of participants with and without the outcome and, if applicable, a summary of the follow-up time. A diagram may be helpful.	Results; study population, para 1 and Figure 1
	13b	D;V	Describe the characteristics of the participants (basic demographics, clinical features, available predictors), including the number of participants with missing data for predictors and outcome.	Results; study population, para 1; Table 1; Table S2
	13c	V	For validation, show a comparison with the development data of the distribution of important variables (demographics, predictors and outcome).	Results; study population, para 1; Table S3
Model development	14a	D	Specify the number of participants and outcome events in each analysis.	Results; classification and multivariable prediction models, para 2; Table 4

	14b	D	If done, report the unadjusted association between each candidate predictor and outcome.	Table 4
Model specification	15a	D	Present the full prediction model to allow predictions for individuals (i.e., all regression coefficients, and model intercept or baseline survival at a given time point).	Table 4
	15b	D	Explain how to the use the prediction model.	Results; classification and multivariable prediction models, para 2; Table S5
Model performance	16	D;V	Report performance measures (with CIs) for the prediction model.	Table 4
Model-updating	17	V	If done, report the results from any model updating (i.e., model specification, model performance).	NA
**Discussion**				
Limitations	18	D;V	Discuss any limitations of the study (such as nonrepresentative sample, few events per predictor, missing data).	Discussion; study strengths and limitations, para 1-5
Interpretation	19a	V	For validation, discuss the results with reference to performance in the development data, and any other validation data.	NA
	19b	D;V	Give an overall interpretation of the results, considering objectives, limitations, results from similar studies, and other relevant evidence.	Discussion; summary of main findings, para 1
Implications	20	D;V	Discuss the potential clinical use of the model and implications for future research.	Discussion; implications for public health practice, para 1-4
**Other information**				
Supplementary information	21	D;V	Provide information about the availability of supplementary resources, such as study protocol, Web calculator, and data sets.	Online
Funding	22	D;V	Give the source of funding and the role of the funders for the present study.	Funding

*Items relevant only to the development of a prediction model are denoted by D, items relating solely to a validation of a prediction model are denoted by V, and items relating to both are denoted D;V. We recommend using the TRIPOD Checklist in conjunction with the TRIPOD Explanation and Elaboration document.

Results

Table S2. Maternal and newborn infant characteristics stratified by post-second day neonatal survival in the validation set*

	Validation Set (n=4,983)				
	Alive (n=4,918)		Dead (n=65)		p-value
	No.	%	No.	%	
Age at positive urine test, yr					
< 20	1,475	30.0	26	40.0	0.200
20 - 29	2,745	55.8	30	46.2	
> 29	698	14.2	9	13.8	
Missing	0	0.0	0	0.0	
Gravidity					
Multigravidae	3,487	70.9	34	52.3	0.001
Primigravidae	1,431	29.1	31	47.7	
Missing	0	0.0	0	0.0	
Maternal body mass index, kg/m2					
BMI ≥ 18.5	2,897	58.9	36	55.4	0.567
BMI < 18.5	2,021	41.1	29	44.6	
Missing	0	0.0	0	0.0	
Living standards index†					
At/below median	2,451	49.8	39	60.0	0.004
Above median	2,460	50.0	25	38.5	
Missing	7	0.1	1	1.5	
Education level					
None	1,408	28.6	18	27.7	0.039
Class 1-4	728	14.8	9	13.8	
Class 5-9	2,497	50.8	36	55.4	
Class ≥ 10	278	5.7	1	1.5	
Missing	7	0.1	1	1.5	
Betel nut chewing‡					
Did not chew	1,569	31.9	20	30.8	< 0.001
Chewed betel nut	3,340	67.9	43	66.2	
Missing	9	0.2	2	3.1	
Tobacco chewing‡					
Did not chew	4,338	88.2	56	86.2	< 0.001
Tobacco chewing	570	11.6	7	10.8	
Missing	10	0.2	2	3.1	
Husband smoking‡					
Did not smoke	1,714	34.9	20	30.8	< 0.001

Husband smoking	3,192	64.9	43	66.2	
Missing	12	0.2	2	3.1	
Experienced prior stillbirth§					
No prior stillbirth	3,185	91.4	29	87.9	0.467
≥ 1 previous stillbirth	298	8.6	4	12.1	
Missing	0	0.0	0	0.0	
Experienced prior abortion§					
No prior abortion	3,087	88.6	28	84.8	0.496
≥ 1 prior abortion	396	11.4	5	15.2	
Missing	0	0.0	0	0.0	
Experienced prior miscarriage§					
No prior miscarriage	3,098	88.9	31	93.9	0.362
≥1 prior miscarriage	385	11.1	2	6.1	
Missing	0	0.0	0	0.0	
Experienced prior infant death§					
No prior infant death	2,590	74.4	19	57.6	0.087
≥1 Prior infant death	722	20.7	11	33.3	
Missing	171	4.9	3	9.1	
Location of delivery‖					
Facility	322	6.5	4	6.2	0.934
Home	4,587	93.3	61	93.8	
Missing	9	0.2	0	0.0	
Infant sex‖					
Male	2,573	52.3	34	52.3	0.999
Female	2,345	47.7	31	47.7	
Missing	0	0.0	0	0.0	
Gestational age at birth, wk					
Mean (sd)	38.8 (2.82)		36.0 (4.72)		< 0.001
≥ 37	3,810	77.5	28	43.1	< 0.001
< 37	908	18.5	30	46.2	
Missing	200	4.1	7	10.8	
Birthweight, kg‖					
Mean (sd)	2.58 (0.40)		1.97 (0.63)		< 0.001
≥ 2.5	2,879	58.5	16	24.6	< 0.001
< 2.5	2,023	41.1	49	75.4	
Missing	16	0.3	0	0.0	
Cyanosis‖					
Absent	4,777	97.1	61	93.8	0.125
Present	117	2.4	4	6.2	
Missing	24	0.5	0	0.0	

Non-cephalic presentation‖					
Absent	4,820	98.0	60	92.3	0.005
Present	96	2.0	5	7.7	
Missing	2	0.0	0	0.0	
Lethargy‖					
Absent	3,254	66.2	36	55.4	0.144
Present	1,640	33.3	29	44.6	
Missing	24	0.5	0	0.0	
Trouble suckling‖					
Absent	4,486	91.2	41	63.1	< 0.001
Present	378	7.7	20	30.8	
Missing	54	1.1	4	6.2	

*Data are n, % unless otherwise specified

†Median living standards index for combined development and validation sets: -.2596556

‡Tobacco exposures measured with respect to the week preceding interview at enrolment

§Data ascertained from pregnancy enrollment among women reporting a previous pregnancy. In the validation set, 3,516 infants were born to mothers in multigravidae, 3,483 remained alive, 33 died.

‖Data ascertained from infant birth assessment or maternal birth assessment.

Table S3. Maternal and newborn characteristics stratified by development and validation set

	Development (n = 14,944)		Validation (n = 4,983)	
	No.	%	No.	%
Age at positive urine test, yr				
< 20	4,592	30.7	1,501	30.1
20 - 29	8,292	55.5	2,775	55.7
> 29	2,060	13.8	707	14.2
Gravidity*				
Multigravid	10,550	70.6	3,521	70.7
Primigravid	4,394	29.4	1,462	29.3
Maternal body mass index, kg/m2*				
BMI ≥ 18.5	8,855	59.3	2,933	58.9
BMI < 18.5	6,089	40.7	2,050	41.1
Living standards index*†				
At/below median	7,459	49.9	2,490	50.0
Above median	7,463	49.9	2,485	49.9
Education level*				
None	4,287	28.7	1,426	28.6
Class 1-4	2,248	15.0	737	14.8
Class 5-9	7,579	50.7	2,533	50.8
Class ≥ 10	810	5.4	279	5.6
Betel nut*‡				
Did not chew	4,584	30.7	1,589	31.9
Chewed betel nut	10,320	69.1	3,383	67.9
Tobacco chewing*‡				
Did not chew	13,171	88.1	4,394	88.2
Chewed tobacco	1,731	11.6	577	11.6
Husband smoking*‡				
Did not smoke	5,180	34.7	1,734	34.8
Husband smoking	9,713	65.0	3,235	64.9
Experienced prior stillbirth§				
No prior stillbirth	9,638	91.5	3,214	91.4
≥ 1 prior stillbirth	894	8.5	302	8.6
Experienced prior abortion§				
No prior abortion	9,331	88.6	3,115	88.6
≥1 prior abortion	1,201	11.4	401	11.4
Experienced prior miscarriage§				
No prior miscarriage	9,373	89.0	3,129	89.0

≥1 prior miscarriage	1,159	11.0	387	11.0
Experienced prior infant death§				
No prior infant death	7,787	73.9	2,609	74.2
≥1 Prior infant death	2,260	21.5	733	20.8
Location of Delivery‖				
Facility	989	6.6	326	6.5
Home	13,926	93.2	4,648	93.3
Infant sex‖				
Male	7,579	50.7	2,607	52.3
Female	7,365	49.3	2,376	47.7
Gestational age at birth, wk‖				
Mean (sd)	38.8 (2.89)		38.8 (2.87)	
≥ 37	11,509	77.0	3,838	77.0
< 37	2,790	18.7	938	18.8
Birthweight, kg‖				
Mean (sd)	2.56 (0.41)		2.57 (0.41)	
≥ 2.5	8,591	57.5	2,895	58.1
< 2.5	6,317	42.3	2,072	41.6
Cyanosis‖				
Absent	14,579	97.6	4,838	97.1
Present	317	2.1	121	2.4
Non-Cephalic Presentation‖				
Absent	14,619	97.8	4,880	97.9
Present	309	2.1	101	2.0
Lethargy‖				
Absent	9,893	66.2	3,290	66.0
Present	4,958	33.2	1,669	33.5
Trouble Suckling‖				
Absent	13,566	90.8	4,527	90.8
Present	1,225	8.2	398	8.0

*Data ascertained at pregnancy enrollment. Missing (%) from derivation and validation cohorts, respectively: Living standards index: 22 (0.1) and 8 (0.2), education level: 20 (0.1) and 8 (0.2), betel nut chewing: 40 (0.3) and 12 (0.2), tobacco chewing 42 (0.3) and 12 (0.2), husband smoking: 51 (0.3) and 14 (0.3).
†Median living standards index for combined derivation and validation sets: -.2596556

‡Tobacco exposures measured with respect to the week preceding interview at enrolment

§Data ascertained from pregnancy enrollment among women reporting a previous pregnancy (n = 10,533 in the derivation cohort, n = 3,516 in the validation cohort). Missing (%) from derivation and validation cohorts, respectively; experienced prior stillbirth 0 (0.0) and 1 (0.0), experienced prior abortion 0 (0.0) and 1 (0.0), experienced prior miscarriage 0 (0.0) and 1 (0.0), experienced prior infant death 486 (4.6) and 174 (4.9)

‖Data ascertained from infant birth assessment or maternal birth assessment. Missing (%) from derivation and validation cohorts, respectively; gestational age at birth 645 (4.3) and 207 (4.2), birthweight: 36 (0.2) and 16 (0.3), cyanosis: 48 (0.3) and 24 (0.5), non-cephalic presentation: 16 (0.1) and 2 (0.0), lethargy: 93 (0.6) and 24 (0.5), trouble suckling: 153 (1.0) and 46 (1.2) Gestational age at birth was calculated with last menstrual period as an estimate for onset of pregnancy.

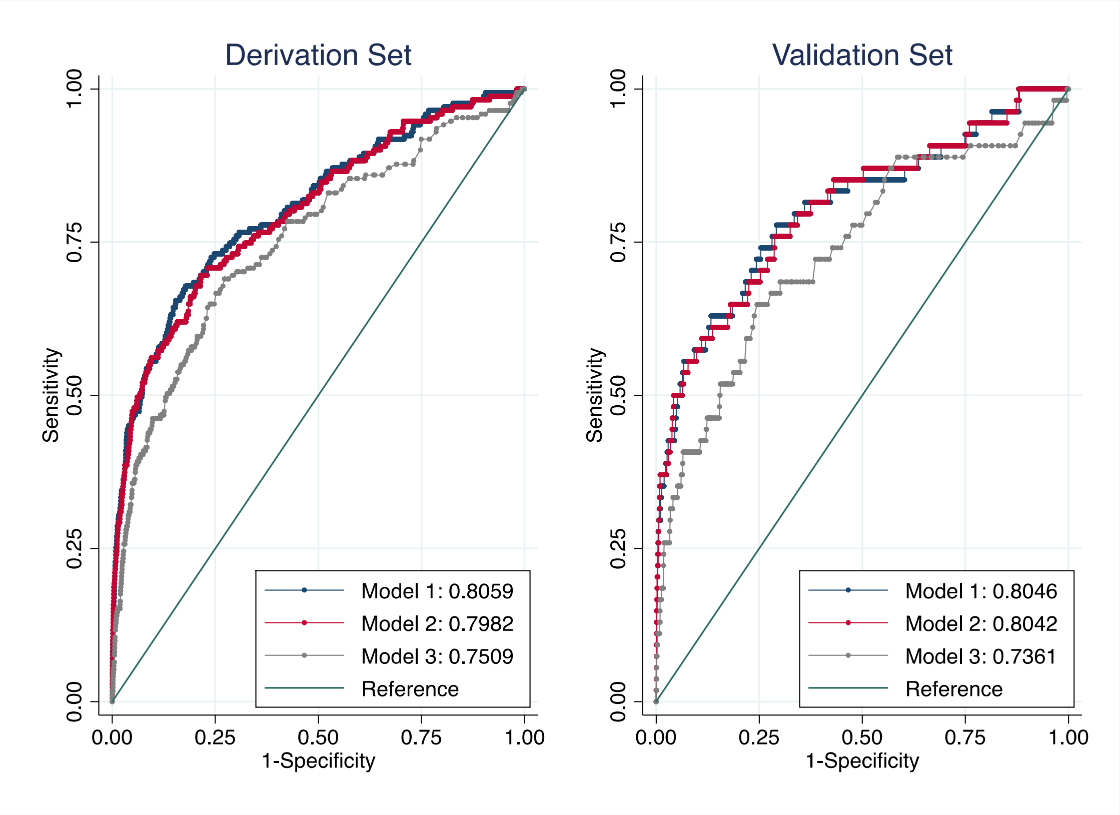

Figure S1: Receiver-Operating Characteristic Curves for Multivariable Logistic Regression Models

Curves constructed using the sample from Model 1 (all predictors and covariates, n = 13,932 in the development set (left) and n = 4,644 in the validation set (right)). Model 2 includes birthweight, gestational age, lethargy, cyanosis, non-cephalic presentation, and poor suckling. Model 3 includes gestational age, lethargy, non-cephalic presentation, and poor suckling. Model AUC is indicated in the legend.

Table S4. Prediction Models for 2-27d Neonatal Mortality and Model Discrimination and Calibration Among Multigravidae

	Univariable	Model A		Model B		Model C		Model D*	
Total number of newborns		9,353		9,812		9,858		10,047	
Number of newborn deaths		93		102		104		103	
**Predictors**	**OR (95% CI)**	**OR (95% CI)**	**β**	**OR (95% CI)**	**β**	**OR (95% CI)**	**β**	**OR (95% CI)**	**β**
Birthweight, kg†									
≤ 1.5	0.00 (0.00, 0.01)	0.00 (0.00, 0.04)	-6.057	0.00 (0.00, 0.06)	-5.558				
> 1.5	0.12 (0.07, 0.19)	0.18 (0.10, 0.33)	-1.701	0.18 (0.10, 0.31)	-1.735				
Gestational age, wk	0.84 (0.81, 0.88)	0.93 (0.88, 0.99)	-0.069	0.94 (0.88, 0.99)	-0.065	0.86 (0.82, 0.90)	-0.151		
Lethargy	2.19 (1.51, 3.18)	1.52 (0.98, 2.36)	0.416	1.47 (0.96, 2.23)	0.382	1.72 (1.15, 2.56)	0.541		
Cyanosis	1.01 (0.25, 4.11)	0.37 (0.05, 2.88)	-1.003	0.75 (0.17, 3.29)	-0.291				
Non-cephalic presentation	3.25 (1.56, 6.74)	2.28 (1.01, 5.16)	0.824	2.07 (0.93, 4.64)	0.730	2.41 (1.13, 5.17)	0.880		
Poor suckling	4.86 (3.14, 7.53)	2.13 (1.21, 3.73)	0.755	2.66 (1.61, 4.39)	0.977	4.04 (2.55, 6.38)	1.396		
≥ 1 prior abortion	1.02 (0.57, 1.82)	1.54 (0.83, 2.86)	0.434					1.29 (0.72, 2.32)	0.256
≥ 1 prior miscarriage	1.16 (0.66, 2.03)	0.79 (0.36, 1.75)	-0.235					0.67 (0.31, 1.45)	-0.401
≥ 1 prior stillbirth	1.68 (0.97, 2.90)	1.59 (0.82, 3.07)	0.463					1.81 (1.00, 3.26)	0.593
≥ 1 prior infant death	2.03 (1.36, 3.04)	1.54 (0.98, 2.42)	0.431					2.03 (1.36, 3.05)	0.709
Intercept			8.160		7.504		0.747		-4.832
**Discrimination and Calibration**									
Area Under ROC Curve (95% CI)									
Derivation		0.78 (0.72, 0.83)		0.77 (0.71, 0.82)		0.70 (0.64, 0.76)		0.60 (0.54, 0.65)	
Validation		0.83 (0.75, 0.91)		0.82 (0.72, 0.91)		0.74 (0.62, 0.85)		0.60 (0.50, 0.70)	
Hosmer Lemeshow χ2 (p)									
Derivation		6.91 (0.55)		6.85 (0.55)		6.32 (0.61)		0.23 (0.89)	
Validation		4.69 (0.91)		8.00 (0.63)		13.95 (0.18)		1.71 (0.79)	

*Hosmer-Lemeshow test performed on 4 quantiles due to limited covariate patterns.

†Birthweight modeled as a linear spline with a knot at 1,500 g.

Table S5. Equations to calculate predictive probability of an individual newborn death using Model 2 and 3

Model	Equation
Model 2*	$\frac{e^{7.787 - 4.853(birthweight \leq 1.5\,kg) - 1.904(birthweight > 1.5\,kg) - 0.097\,(gestational\ age) + 0.426(lethargy) + 0.326(cyanosis) + 0.726(non-cephalic\ presentation) + 0.947(poor\ suckling)}}{1 + e^{7.787 - 4.853(birthweight \leq 1.5\,kg) - 1.904(birthweight > 1.5\,kg) - 0.097\,(gestational\ age) + 0.426(lethargy) + 0.326(cyanosis) + 0.726(non-cephalic\ presentation) + 0.947(poor\ suckling)}}$
Model 3*	$\frac{e^{2.707 - 0.203(gestational\ age) + 0.668(lethargy) + 0.904(non-cephalic\ presentation) + 1.381(poor\ suckling)}}{1 + e^{2.707 - 0.203(gestational\ age) + 0.668(lethargy) + 0.904(non-cephalic\ presentation) + 1.381(poor\ suckling)}}$

*Model 2: For "(birthweight ≤ 1.5 kg)", enter birthweight in kg if below 1.5 kg or 1.5 if greater than 1.5 kg. For "(birthweight > 1.5 kg)" enter the difference between birthweight and 1.5 kg. If the baby is below 1.5 kg, then enter 0. For "(cyanosis)", enter 1 if any part of the baby's body was blue at birth or enter 0 if no part of the baby's body was blue at birth. Model 2 and 3: For "(gestational age)" enter the gestational age of the baby at birth. For "(lethargy)", enter 1 if the baby either did not move at birth, moved weakly at birth, did not cry at birth, or cried weakly at birth; or enter 0 if baby moved strongly and cried strongly at birth. For "(non-cephalic presentation)" enter 1 if any part besides the head came out first or enter 0 if another part besides the head came out first. For "(poor suckling)", enter 1 if the baby is having trouble sucking or feeding in the two days following birth, or 0 if the baby is not having trouble sucking or feeding. Multiply the probability by 100 to ascertain the risk in percentage points.

References

1 Moons KGM, Altman DG, Reitsma JB, *et al.* Transparent reporting of a multivariable prediction model for individual prognosis or diagnosis (TRIPOD): Explanation and elaboration. *Ann Intern Med* 2015;**162**:W1–73. doi:10.7326/M14-0698

2 Collins GS, Reitsma JB, Altman DG, *et al.* Transparent Reporting of a multivariable prediction model for Individual Prognosis or Diagnosis (TRIPOD): the TRIPOD statement. *Ann Intern Med* 2015;**162**:55–63. doi:10.7326/M14-0697
